# Labour force activity after 65: what explain recent trends in Denmark, Germany and Sweden?

**DOI:** 10.1007/s12651-017-0223-7

**Published:** 2017-03-20

**Authors:** Mona Larsen, Peder J. Pedersen

**Affiliations:** 1 SFI – Danish National Centre for Social Research, Herluf Trolles Gade 11, DK-1052 Copenhagen, Denmark; 2 SFI – Danish National Centre for Social Research, Aarhus University, Aarhus, Denmark; 3 IZA, Bonn, Germany

**Keywords:** Employment, Older workers, Program changes, Education, Health, I15, I25, J14, J26

## Abstract

In most OECD member countries labour force attachment, has increased in recent years not only in the age groups 60–64 years but also among people 65 years and older. Focus in this paper is on the trend in older workers’ labour force participation in Denmark, Germany and Sweden since 2004. Main emphasis is given to people aged 65–69 years eligible for social security retirement programs from age 65. The gender aspect is included to accommodate different trends for women and men. To explain country differences in trends, the importance of changes in retirement policies of relevance for this age group and cohort relevant changes in education and health is examined and discussed. Further, country differences in the impact from education and health is examined. Results show that the largest increase in labour force participation among people aged 65–69 years has taken place in Sweden following by Germany, while the increase in Denmark is rather small. While the increase in Germany mainly seems to be a result of policy reforms, the increase in Sweden appear to be a result of a combination of policy changes and an increasing educational level. Financial incentives seem most important in Germany and only of minor importance in Denmark, where policy changes directed towards individuals above the age of 65 appear to have been too small so far to affect retirement behaviour significantly.

## Zusammenfassung

In den meisten OECD-Mitgliedsländern ist in den letzten Jahren nicht nur die Beschäftigung in der Altersgruppe der 60–64-jährigen, sondern auch bei Personen ab 65 Jahren gestiegen. Dieser Artikel analysiert die Arbeitsmarktbeteiligung Älterer in Dänemark, Deutschland und Schweden seit 2004. Das Hauptaugenmerkt liegt dabei auf Menschen in der Altersgruppe der 65 bis 69-jährigen. Geschlechtsspezifische Unterschiede werden berücksichtigt, um unterschiedliche Entwicklungen für Frauen und Männer erfassen zu können. Um länderspezifische Unterschiede erklären zu können, werden wichtige Veränderungen in den Rentenprogrammen, die für diese Altersgruppe relevant sind, sowie Änderungen im Bildungsniveau und Gesundheitszustand dieser Alterskohorte untersucht und diskutiert. Weiterhin werden länderspezifische Unterschiede beim Einfluss von Bildung und Gesundheit untersucht. Die Ergebnisse zeigen, dass die größte Steigerung der Arbeitsmarktteilnahme unter den 65 bis 69-jährigen in Schweden stattgefunden hat, gefolgt von Deutschland, während die Steigerung in Dänemark eher gering ausfällt. Während der Anstieg in Deutschland vor allem ein Ergebnis politischer Reformen zu sein scheint, ist der Anstieg in Schweden auf eine Kombination aus politischen Reformen und einem gestiegenen Bildungsniveau zurückzuführen. Finanzielle Anreize scheinen in Deutschland den größten Einfluss gehabt zu haben. Demgegenüber waren die Rentenänderungen für über 65-jährige in Dänemark zu gering, um deren Arbeitsmarktbeteiligung signifikant zu beeinflussen.

## Introduction

A common feature for many OECD countries is a remarkable increase in labour force participation rates of individuals aged 60 and above since the mid-1990s. This is a reversal of a general long run trend towards lower average retirement ages, see e. g. Larsen and Pedersen (2013) and Anxo et al. (2012). In many countries, the increase in labour force participation rates among older workers continued even after the great recession beginning in 2008 – a period in which non-employment rates have increased markedly among younger people (OECD 2014a). Increasing labour force participation rates for older workers is important in the light of the need in most rich OECD countries of adapting to the increasing share of the population being 65 and older until the middle of the century. These demographic prospects are the net outcome of a low level of fertility combined with an increase in longevity. The purpose of this paper is to examine and discuss some of the main factors contributing to explain the recent increase in employment rates for individuals aged 65–69 years. Most policy changes have until recently focussed on making early retirement less attractive, thus contributing along with other factors to explain the increase in labour force participation among people 60–64 years old. As summarized below, policy measures have also been introduced to make it more attractive for the 65–69 years old to stay in the labour force. However, while future changes have been announced in the age of eligibility for old age pension, in the years we analyse here people are eligible from age 65. This creates a setting where preferences and options are more important for the decision to keep on working for this age group than for people aged 60–64 years. Another obvious argument for looking at the 65–69 years old is the fact that the potential for increasing employment is much bigger here due to the fairly low initial level.

One contribution of this paper is to add to the literature on determinants of retirement after the age of eligibility for old age pension. Further, in contrast to most research in this area, we conduct a cross-country comparison of the main factors behind the recent increase in labour force participation. Finally, while most recent research in this area is based on US data, our focus is on three European countries, namely Denmark, Germany and Sweden.

Denmark, Germany and Sweden face the same demographic challenge. However, they also differ in several ways making it relevant to analyse how differences in the national background influences the common outcome, the employment rate among 65–69 years old people. In the terminology of Esping-Andersen (1990) Germany is a continental or conservative type welfare state while Denmark and Sweden belong to the social democratic type of welfare state. Although Denmark and Sweden share a number of similarities, i. e. fairly generous welfare states based on a large public sector, high taxes, mostly universal benefit programs, and high labour force participation for women, they also differ at the same time in a number of respects such as industrial structure, cyclical experience during recent decades and retirement policies. Further, the selected countries differ in a number of other ways that might – directly or indirectly – affect the country specific trend in labour market activity after age 65. For instance, Germany faces a larger demographic challenge than Denmark and Sweden. While the old age dependency ratio is estimated to increase by 30 percentage points in Germany from 2012 to 2050, a much smaller increase is estimated for Denmark and Sweden, namely 10–13 percentage points (OECD 2015). In addition, the net pension replacement rate for the average earner is higher in Denmark (66%) and in Sweden (64%) than in Germany (50%), (OECD 2016). Finally, the average economic growth was about 1 percentage point higher in Sweden than in Denmark and Germany during the period 1996–2013, see Fig. 6 in the Appendix.

**Fig. 1:**
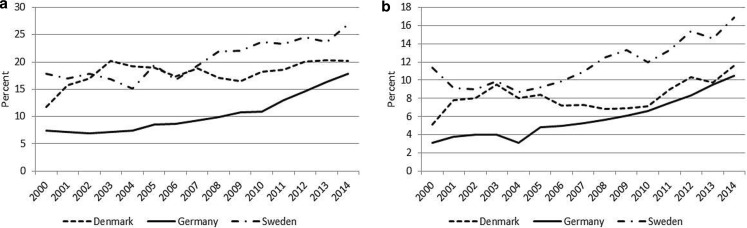
Labour force participation rates, for individuals aged 65–69 years, separately for a men, and b women, 2000–2014. Percent. (Source: Eurostat)

**Fig. 2:**
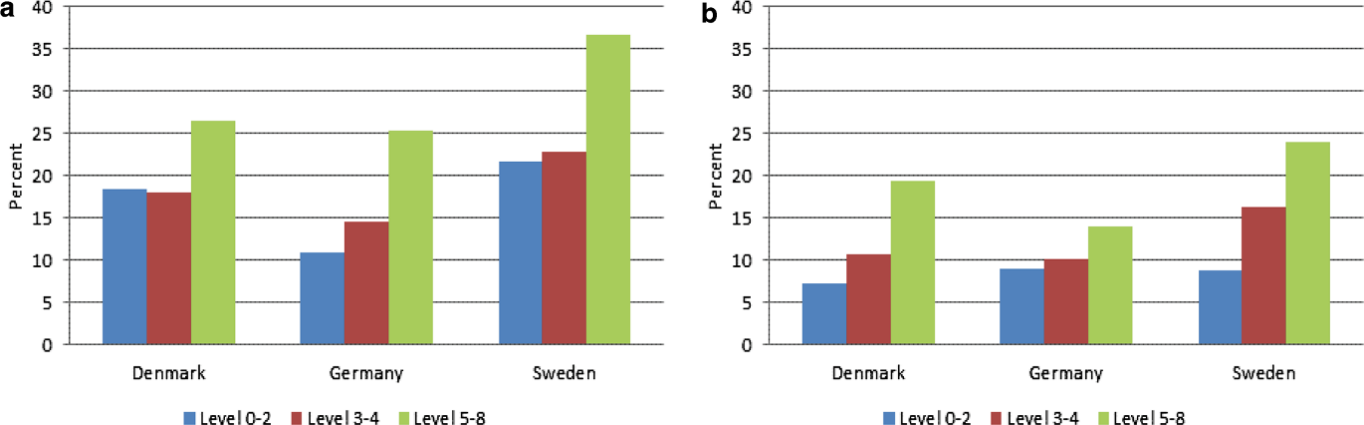
Employment rate by educational level^1)^ for individuals aged 65–69 years, separately for a men, and b women, 2014. Percent. (Source: Eurostat (variable: lfsa_ergaed).^1)^ISCED levels are split into three groups: Level 0–2, level 3–4 and level 5–8)

**Fig. 3:**
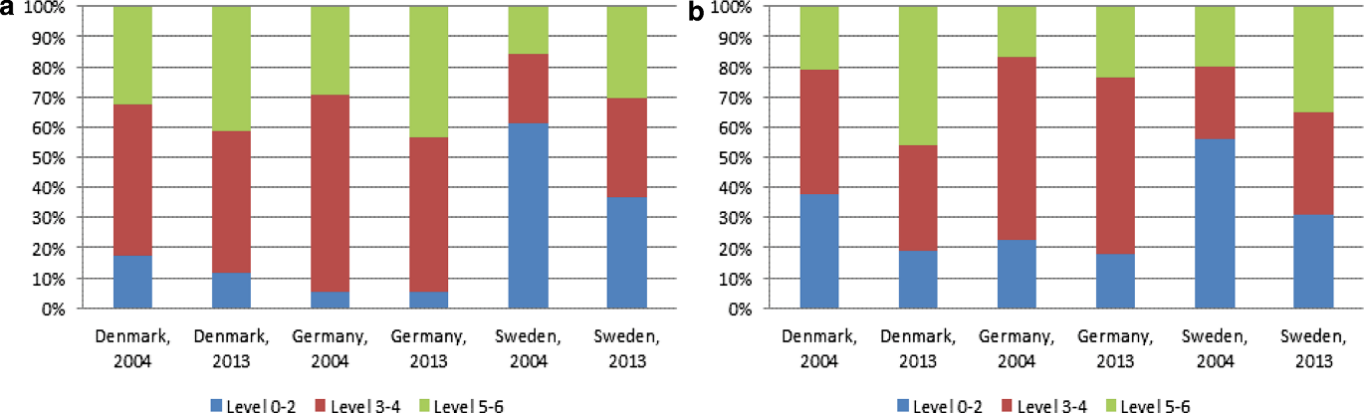
Educational level^1)^ for individuals aged 65–69 years, separately for a men, and b women, 2004 and 2013. Percentage points. (Source: Own calculations on SHARE data (wave 1 and wave 5). ^1)^ISCED levels are split into three groups: Level 0–2, level 3–4 and level 5–6)

**Fig. 4:**
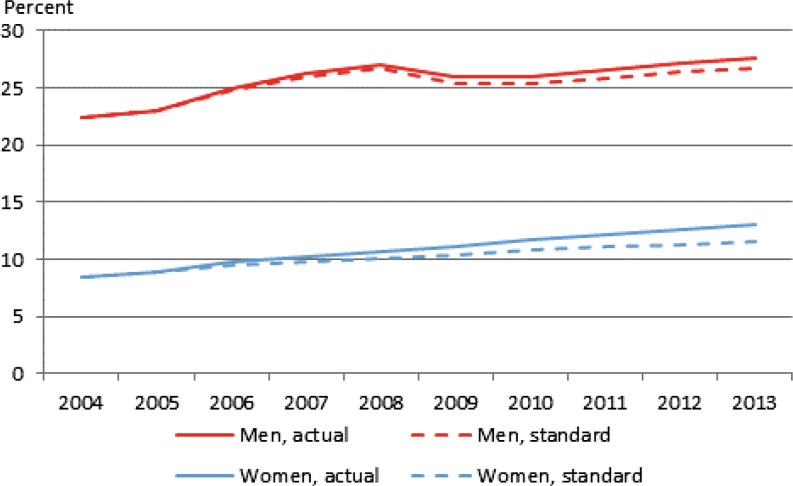
Labour force participation rates for men and women aged 65–69 years, actual and standardized rates, Denmark, 2004–2013. Percent. (Source: Danish register data)

**Fig. 5:**
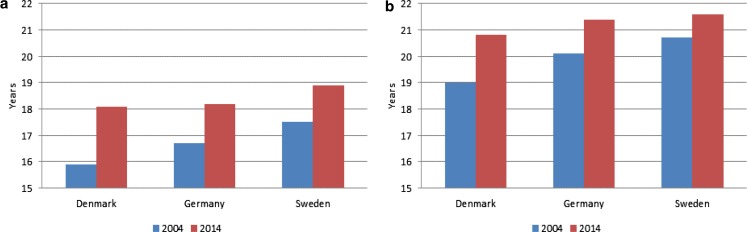
Life expectancy at age 65, separately for a men and b women, 2004 and 2014. Years. (Source: European Commission 2016)

## Review of the literature

OECD (2013) presents some of the factors assumed to explain the strong labour market performance of older workers continuing also during the great recession beginning in 2008. The economic incentives to stay longer in the labour market have become stronger. This is a reflection of many policy changes intending to reduce the implicit tax on continued work at older ages. At the same time each new cohort of older workers are better educated and by available indicators they also appear to be in better health, both potential factors in explaining longer working lives. Another factor is the changing nature of jobs over time, i. e. hard physical work has become much less important due to technological changes and the shift to jobs in the service sector. While these changes have improved the possibilities for older workers to continue in the job, other job related factors like stress and fast changes in technology and job functions might work in the opposite direction.

A number of recent studies focus on explaining the increasing employment trend for older workers. According to Gruber and Wise (1999), a high implicit tax on continued work from a specific age was one of the main explanations of the decline in average retirement ages until a reversal of this trend in the late 1990s in most OECD countries. Maestas and Zizzimopolous (2010) discuss this reversal in older individuals’ labour force participation in relation to several possible explanatory factors: secular changes in the skill composition of the labour force, technological change removing the very physically demanding jobs along with changes in the incentive structure inherent in the social security programs and in employer provided pensions. Friedberg (2007) focuses on the declining labour force participation rate until the mid-1990s, followed by a levelling off and a trend upwards for older workers in more recent years. As potential explanations of this profile, she points to changes in social security program incentives, in employer provided pensions and in health care. Burtless (2013) and Blau and Goodstein (2010), however, attach more importance to increasing educational attainment than to changes in the retirement income system. Higher educational levels tend to increase labour force participation because education increases return to work, and because education might increase task complexity and work autonomy and, thereby, increase the intrinsic value of work (OECD 2013). For instance, using Danish data Larsen (2008) finds that influence on the job and being able to organise own work increases planned retirement age for older men. The emphasis in Cai (2010) is on the positive impact from improved health on continued labour force participation. Hence, much of the available empirical evidence suggests that poor health causes workers to retire earlier (see e. g. Bound 1991; Dwyer and Mitchell 1999; McGarry 2004). Finally, focusing on the U.S., Canada, and the U.K., Schirle (2008) finds that a substantial share of the increase in labour force participation among older married men may be explained by the increase in recent decades in the participation rates of their wives because wives’ leisure time is complementary to the leisure time of their husbands.

Two recent German studies focus on retirement or continued work for the whole age group 60 years and older. Using GSOEP data, Pfarr et al. (2015) find a strong relative increase in labour force participation both in the age group 60–64 and in the 65+ group. The findings by Pfarr et al. (2015) suggest that the whole increase in employment in the 65+ group occurs in the years 2001–2004, i. e. there is no impact per se from the great recession beginning in 2008. In estimations, Pfarr et al. (2015) find that economic incentives seem to play a small role for the choice between retirement or continued work. Tolan (2015) is a brief discussion of the impact from more flexible retirement options. For the 65+ group, Tolan (2015) points to the importance of whether employers have to pay social contributions for workers in this age group. Three other recent contributions by German authors, Wübbeke (2013), Möhring and Bennett (2015) and Bönke et al. (2016) are interesting analyses in the area of early retirement, leaving the labour force before age 65. The focus below is, however, as mentioned on those who continue work beyond age 65.

Using US data, Beehr and Bennett (2015) analyse bridge jobs as an option in the transition to full retirement. US data are also used in Cahill et al. (2015) where the topic is the potential impact from macroeconomic factors on several forms of behaviour around the standard retirement age, i. e. bridge jobs, phased retirement in a present job or un-retirement where a person returns to the labour force after a period as retired.

A number of studies focus on the motivations for continuing work or retiring using qualitative data. Griffin and Hesketh (2008) work with two samples consisting of about 1000 individuals in a pre-retirement sample and about 1000 in a sample of retired people. They find education to be especially important for continuing paid work while gender, health and satisfaction with being retired are of special importance for volunteer work after retirement. Dingemanss and Henkens (2014) focus on subjective well-being in different exit routes from the labour market, relative to staying in work. Furunes et al. (2015) are working with data from qualitative interviews in Norway collected in three waves of individuals 58 years and older. They find non-economic motives and health to be more important for the decision of continuing to work or not, than financial incentives. Fassbender et al. (2016) are using data from a German survey in an analysis with focus on the “meaning of work” in four different dimensions. Attaching positive feelings to those “meanings of work” are predictors of post-retirement work. Besides, self-assessed health is found important for post-retirement work.

While the studies mentioned above focus mainly on supply oriented factors, the demand side of the labour market is also expected to be important. A supply driven behaviour must obviously meet a demand, i. e. employers that keep older workers, even in a setting of a very deep recession. A discussion of the demand aspect can for instance be found in Dalen et al. (2010). However, in line with much of the existing literature on older workers, we concentrate on supply oriented explanations of the recent trends in labour market participation for the age group 65 to 69 years. We focus on the impact from changes in a) retirement policies, b) education, and c) health. We examine these explanations separately for men and women.

In the following, Sect. 3 contains a survey of the types of data and the approach used in the subsequent descriptions and a description of the aggregate development in labour force participation in the three countries. Next, we consider and discuss the importance of changes in retirement policies (Sect. 4), education (Sect. 5), and health (Sect. 6) in each of these countries. In Sect. 7, we conduct regression analyses to examine country differences in the impact of education and health, while controlling for age. Finally, Sect. 8 concludes the paper.

## Data sources, method and recent trends in labour force participation

In this section, we first introduce the data sources and method used in this study. Next, we document the trends in older workers’ labour force participation in each of the selected countries.

### Data sources and method

The initial aggregate overview of labour force participation in the 60 and older group presented for the three countries is based on survey information obtained from Eurostat. These data apply in principle ILO criteria to the classification of individuals in relation to the labour force. In this way individuals may have entered an early or normal age retirement program but still be working to an extent where they are classified as being in the labour force. Furthermore, individuals can enter a retirement program, have a spell completely without any work activity and then re-enter the labour market to a job, the so called un-retirement behaviour.

Labour force participation in the 60 and older group is expected to depend crucially on education and health, both factors changing over time with still more recent cohorts of workers, cf. Blau and Goldstein (2010), Burtless (2013) and Cai (2010). From EUROSTAT we obtain information about employment rates by educational level in 2014. Further, we use information from the Survey of Health, Ageing and Retirement in Europe (SHARE) about labour force participation and educational level in 2004 (wave 1) and 2013 (wave 5) 1for the three countries. We use these data to illustrate how individuals aged 65 to 69 years are distributed on educational level in these two years. Further, we make simple shift share analyses of the impact of the increasing educational level (split into three levels, *i* = 1–3) on labour force participation from 2004 to 2013. We calculate the impact as follows: First, we calculate a standardised labour force participation rate (LFP_S_) in 2013 by 1) multiplying the actual labour force participation rate (LFP_A_) for each of the three educational levels (i) in 2013 by the share of individuals (*n*) at each educational level (i) in 2004 and by 2) adding these three products into one figure, see Eq. 1. This figure shows what the labour force participation rate would have been if the distribution on educational level was the same in 2013 as in 2004.

1 $$LFP_{{S}_{2013}}=\overset{i=3}{\underset{i=1}\sum }LFP_{{A}_{2013}}^{i}*n_{2004}^{i}$$

Second, by using Eq. 2, we obtain the share of the actual change in the labour force participation rate from 2004 to 2013 that can be explained by an increase in the educational level:

2 $$\text{Impact}_\text{Education}=\frac{\left (LFP_{{A}_{2013}}-LFP_{{A}_{2004}}\right )-\left (LFP_{{S}_{2013}}-LFP_{{A}_{2004}}\right )}{\left (LFP_{{A}_{2013}}-LFP_{{A}_{2004}}\right )}$$

Because of small sample size in wave 1 of SHARE for the age group 65–69 years in Denmark, the separate calculations for men and women in particular must be interpreted with caution.^2^ To supplement the calculations for Denmark on SHARE data, we also conduct similar shift share analyses on aggregate Danish register data for each year in the period 2004 to 2013. The data cover the total population in the age group 65 to 69 years and therefore, these data give us more reliable calculations for Denmark.

To evaluate the extent of changes in health, we use the European Community health indicators (European Commission 2016) about life expectancy at age 65 for 2004 and 2014. Finally, to examine country differences in the impact on labour force participation from education and health while controlling for age, we run probit regressions. For this purpose, we use SHARE data from 2013, i. e. from wave 5 of SHARE.

### Recent trends in labour force participation

In this section, we compare the recent trends in labour force participation among individuals aged 65–69 years in Denmark, Germany, and Sweden. The country specific profiles for this age group for the period 2000 to 2014 are shown in Fig. 1 separately for women and men. We find that the increase in the labour force participation rates mainly occurs from around 2004 in Germany and Sweden, while in Denmark we see a stationary level until 2009 followed by an increase.^3^ Consequently, to fulfil our purpose of examining some of the main factors behind the increase in labour force participation for individuals aged 65–69 years, we focus on explaining the development in the period from 2004 and on.

The largest increase (in absolute terms) from 2004 to 2014 is found in Sweden for both men and women followed by Germany, while the increase is much smaller in Denmark. Comparing men and women within each country, we find a larger increase (in absolute terms) for men than for women in Germany and Sweden, while the opposite is found in Denmark. In 2014, we find the highest labour force participation rate in Sweden and the lowest in Germany.

## Changes in retirement policies

Policy changes and reforms in the areas of pensions and retirement are expected to have a significant potential impact on the increase in labour force participation rates. Since the turn of the millennium, a number of policy changes have been enacted in this area of relevance for the age group 65–69 years in Denmark, Germany and Sweden. Here, we include a brief survey of the reforms in each of these countries and present existing evidence of their impact on labour force participation among individuals aged 65–69 years.

### Denmark

The Danish pension system is a combination of public (pay-as-you-go, defined benefits) and labour market (funded, defined contribution) pensions. Labour market pensions have been subject to rising coverage and contribution rates since the early 1990s. The Danish system effectively prevents old age poverty and ensures fairly high replacement rates for most pensioners (Andersen 2015).

The main Danish strategy to cope with an ageing population is beginning in 2019 to link the age of eligibility in the main social security program for old age pension to the future changes in expected life time. Until now, emphasis has been on tightening access to the main program for non-health related early retirement, the so-called Post Employment Wage (PEW). Conditional on a sufficient long period of membership in an Unemployment Insurance fund and on being in the labour force until entry to the program, workers are eligible for PEW up to five years before the normal retirement age, which today is 65. For a description of recent and future changes of the PEW program, see e. g. Larsen and Pedersen (2013) and Midtsundstad and Bogen (2013).

The main retirement program in the area of Social Security is Old Age Pension (OAP), a pay-as-you-go program for which everybody today is eligible from age 65. Eligibility to the full amount of OAP requires 40 years of residence. A few policy changes have been implemented in order to increase the incentives to continue working after the normal retirement age (see e. g. OECD 2015; Midtsundstad and Bogen 2013; Larsen and Pedersen 2013; Ministry of Finance 2011). Surprisingly, however, the normal retirement age was lowered gradually from 67 in 2004 to 65 in 2006 to reduce expenditures for retirement for the 65–66 years old. Take up of PEW was very high in this group and benefits under the PEW program are significantly higher than in OAP. At the same time, the possibility to defer OAP on actuarial terms from 65 to 75 conditional on working at least 1500 h a year (29 h per week on average) was implemented. This working criterion was lowered to 1000 h a year (19 h a week on average) in 2008 and to 750 h a year (15 h a week on average) in 2014. In 2008, means testing of supplementary OAP against income from work was reduced. In 2008, mandatory retirement at age 70 was abolished for most groups of public sector employees. Further, it was decided to increase the normal retirement age gradually from 65 in 2019 to 67 in 2022. After 2022 this age will be indexed to increases in expected life time.

Provisions of the social security system play an important role in determining retirement behaviour in Denmark (see .e. g. Bingley et al. 2004). However, available evidence suggests that the impact in Denmark of recent policy changes is of minor importance for postponing retirement till after age 65 (Amilon and Nielsen 2010; Larsen and Ellerbæk 2012). Mainly men, highly educated and people holding advanced positions have chosen to defer their OAP – individuals that wanted to continue working after the official retirement age and, in many cases, might have done so without the opportunity to defer their pensions. Similarly, the impact of reducing means testing of supplementary OAP against income from work seems to be limited. Yet, people in the potential target group were not well informed about either the possibility to defer OAP or the reduction of means testing of supplementary OAP from income from work. More importantly, however, among employed Danes in the age group 65–75 the vast majority state that they work because they have preferences for continued work, while only 30% also mention economic reasons as their motive for working (Larsen et al. 2011), cf. also the results of the analyses focusing on motivation discussed in Sect. 2. All in all, the available evidence suggests that the (small) increase in labour force participation rates among Danes aged 65–69 years are mainly attributed to others factors than recent policy changes directed towards this age group.

### Germany

Public pensions in Germany are designed to extend the relative standard of living achieved during work life to the time after retirement. Thus, pensions only have few redistributive properties, except a minimum pension at the social assistance level, see e. g. Börsch-Supan et al. (2015). Therefore, the German pension system can be considered more as “retirement insurance” than as “social security” (Börsch-Supan and Wilke 2006). In the light of a particularly dramatic change in the age structure of the German population in the future, several cost cutting reforms have been implemented since 1992 to ensure a sustainable pension system. According to Börsch-Supan et al. (2015), the consequences for the German households of these reforms are: a) The generosity of the state-financed public pensions has been reduced and will decrease further thereby lowering income from the pay-as-you-go pillar; b) the statutory retirement age will be increased gradually, and c) additional occupational and private pillars in the pension system have been strengthened. In particular, tax incentives create additional motives to save privately for retirement.

Two specific reform elements are directed to reduce the incentives to retire early. First, actuarial adjustments of benefits to the retirement age have been implemented in the period 1998–2006. Second, the normal retirement age will be increased from 65 to 67 (being fully effective in 2029), the age limit for old age pension for disabled is shifted to 65, and a specific old age pension for women is effectively phased out. However, a partial setback to these changes in the age limits was the introduction in 2014 of new temporary early retirement pathways for specific groups allowing them to retire at age 63 (Börsch-Supan et al. 2015).

German pension reforms seem to a large extent to contribute to explain the increase in labour force participation rates among individuals aged 65–69 years. According to a survey of the existing evidence on the impact from these reforms (Börsch-Supan et al. 2015), Germans have reacted to the changes in incentives implemented and raised their actual retirement ages. Hence, financial incentives are found to matter a great deal in Germany. In particular, the labour supply decision of German old age pension recipients above the age of 65 is found to be significantly motivated by financial considerations. Eschelbach (2011) finds a significant negative relationship for this group between non-labour income and the probability of working, a relationship that is only present for low income groups. Similarly, Hochfellner (2013) shows that the probability of holding a job after age 65 is declining with increasing pension incomes.

### Sweden

The main purpose of the Swedish OAP system is to compensate for income loss from work (Olsen 2002). In addition to OAP, most people in Sweden also have occupational pensions, which are becoming increasingly important over time. The OAP system was changed considerably in the period 1999 to 2003. While the old system was a pay-as-you-go defined benefit pension system, the new system is a mixture of a notional defined contribution pay-as-you-go pension system and a fully funded pension scheme with individual accounts. In the new system, lifelong accrual is implemented. Payments are longevity adjusted and adjusted according to the development in the Swedish economy. Individuals with small or no pension claims receive a guaranteed pension. There is no normal retirement age, but a minimum age of withdrawal at 61. Partial pension is abolished. Instead, it is possible to combine work and pension from age 61. Economic incentives to delay payments of pensions are implemented implying a gain for each month pension payments are delayed from age 61 and on. Unlimited deferral of retirement is made possible in the pension system, but the employer’s consent is required after the age of 67 (before 2001/2003, this age limit was 65 years^4^). Partial pension tied to occupational pension schemes was implemented in 2003 for state employees and in 2007 for local government employees. Further, two different labour tax credits were introduced for workers aged 65 and above in 2007. First, with the purpose of stimulating labour supply, an earned income tax credit was introduced to all workers that was substantially larger for those older than 65. The size of this tax deduction increased gradually from 2007 and on. Second, to compensate for e. g. productivity declines or workplace accommodations, a payroll tax credit aimed at stimulating demand was introduced for workers aged 65 or above (Johansson et al. 2014; Midtsundstad and Bogen 2013; Laun 2012; OECD 2012; Lindquist and Wadensjö 2009).

Although Sweden has introduced much stronger economic incentives in their old age pension system than e. g. Denmark (Midtsundstad and Bogen 2013), these changes only seem to have a moderate impact on labour force participation among individuals aged 65–69 years. Certainly, Klevmarken (2010) assumes that increases in labour force participation after 2006 is related to the introduction of age-targeted tax credits in 2007. However, preliminary results in Laun (2012) suggest that the changes in retirement behaviour induced by these tax credits are moderate. Further, despite considerable changes of the age thresholds in the pension system, most people still take up a pension and retire at age 65 (Albin et al. 2015; Lindquist and Wadensjö 2014).

## Changes in education

To illustrate the importance of the educational level for labour force participation, we first show the employment rate^5^ by educational level in 2014 for individuals aged 65 to 69 years in each of the three countries in Fig. 2. As expected, the highest employment rates are found for highly educated individuals – this result applies for both men and women in all three countries. For women, the employment rate increases by educational level in Denmark and Sweden, while in Germany, these rates seem to be more similar across educational levels. We examine country differences in the impact of the educational level on labour force participation in more detail in Sect. 7 below.

Part of the explanation of the increase in the labour force participation rates might thus be that the educational level has increased among the 65–69-year-olds in the three countries. Using SHARE data, we first illustrate the increase in the educational level in this age group. Next, we show results of a shift share analysis to illustrate the extent in which the increasing educational level can explain the increase in the labour force participation rate in each of the three countries.

In all three countries, the average educational level in the age group 65–69 years has increased from 2004 to 2013, see Fig. 3. Looking at Fig. 3 it seems however also that the measurement of education in SHARE differs between the three countries. As a consequence, we can compare by gender in each of the countries and we can compare the composition in 2004 and 2013 for each country. We can however not compare across countries, see e. g. the extreme differences regarding ISCED 0–2 between Sweden and the other two countries. Overall, it seems that the average educational level increases more for women in Denmark and Sweden than in Germany.

The results of our shift share analysis based on SHARE data are shown in Table 1. These results suggest that the increase in the labour force participation rate to a smaller degree can be explained by an increase in the educational level in Germany than in Denmark and in particular in Sweden. Comparing the impact for men and women in each country, the results suggest that the increasing labour force participation rate to a larger degree is explained by an increasing educational level for women than for men in Denmark and Sweden, while the opposite seems to be the case in Germany.

**Table 1:** The share of the change in the labour force participation rate from 2004 to 2013 for men and women aged 65–69 years that can be attributed to an increase in the educational level

	Denmark	Germany	Sweden
All	0.23	0.14	0.27
Men	0.09	0.23	0.18
Women	0.62	0.02	0.42

Source: Own calculations on SHARE data (wave 1 and wave 5)

The gender difference in Denmark seems to be rather large. However, as a consequence of small sample size in 2004, the results based on SHARE data for Denmark must be interpreted with caution, see also Sect. 2. Therefore, as a supplementary analysis, we have also conducted the shift share analysis based on register data for Denmark. These data enable us to calculate the standardized rate for each year from 2005 to 2013 for men and women respectively, see Fig. 4.

The results in Fig. 4 confirm that the increasing educational level is more important for women than for men in Denmark. Comparing 2004 and 2013, we find that 31% of the increase for Danish women in this period and 18% of the increase for Danish men is explained by an increasing educational level. Comparing these results to the results for Denmark based on SHARE data, these figures seem to be in line with the number for “all” in Table 1. At the same time, these figures suggest that the very large difference between the impact for men and women suggested by the calculations based on SHARE data is a result of the small sample size.

## Health changes

A perfect objective measure of the level of and changes in health over time is not available. Mortality and life expectancy at specific ages are approximations to such an objective measure that can be used in empirical analyses. Self-reported health is a useful subjective indicator. In this section, we compare the level of life expectancy at age 65 in the three countries and how it has developed between 2004 and 2014.

We find for men that life expectancy at age 65 is at about the same level in Denmark and Germany in 2014, while it is higher in Sweden, see Fig. 5. For women, the life expectancy is lowest in Denmark and at the same slightly higher level in Germany and Sweden.

We find the largest increase in life expectancy from 2004 to 2014 in Denmark for both men and women implying that Danish men are catching up with the life expectancy for German men in this period. Similarly, Danish women are approaching the life expectancy of both German and Swedish women. According to European Community Health Indicators, European Commission (2016), the increase in life expectancy is followed by an increase in the number of healthy life years at age 65. In the next section, we examine country differences in the impact from the alternative measure, self-reported health – along with the impact from age and education – on labour force participation by use by probit estimations.

## Regression analyses

In this section we extend the descriptions above of the development in the most recent decade with results from a number of regression analyses for the three countries using data from wave 5 of SHARE collected in 2013. The dependent variable in the regressions is set at 1 if the individual is in the labour force, 0 otherwise. As explanatory variables in the probit estimations we enter age as a control capturing elements in retirement policies, cf. the discussion above, along with an eventual age gradient in the financial incentive to retire. We further include education and self-assessed health from SHARE. We include all respondents 65–69 years old and all estimations are made separately for men and women.

The results for the first specification are shown in Table 2 reporting marginal effects in probit estimations including age, years of education and self-assessed health reported on a scale from 1 as excellent to 5 as poor.

**Table 2:** Marginal effects in Probit estimations on being in the labour force, 65–69 years old, separately for men and women, SHARE W5, Denmark, Germany and Sweden

	Men			Women		
	DK	DE	SE	DK	DE	SE
Age	−0.044*** (−6.52)	−0.058*** (−11.43)	−0.060*** (−10.86)	−0.029*** (−6.09)	−0.009*** (−2.50)	−0.052*** (−12.26)
Years of education	0.003 (1.44)	0.004*** (2.74)	0.006*** (2.92)	0.004*** (2.87)	0.004*** (3.12)	0.008*** (5.37)
Self-assessed health	−0.039*** (−4.40)	−0.054*** (−8.71)	−0.029*** (−4.21)	−0.031*** (−4.91)	−0.025*** (−4.61)	−0.019*** (−3.77)
Pseudo R^2^	0.034	0.2041	0.0741	0.0761	0.0686	0.1324
No. of obs	1765	2000	2273	1816	1935	2760

**Significance 5%, ***Significance 10%

The age coefficient is found to be negative as expected and highly significant. In Denmark and Germany the age gradient seems to be steeper for men than for women, while in Sweden the gradient by gender is at the same level. Years of education has a significant positive coefficient except for men in Denmark^6^. Finally, as expected, self-assessed health has a significant negative coefficient in all cases with a slightly higher gradient for men then for women.

The specification in Table 2 assumes a continuous impact from both age and education in years on labour force participation. To test this assumption we define dummy variables for single year ages from 65 to 69. Further, we define three educational variables as dummies, i. e. “low” for years of education 9 years or lower, “medium” for number of years higher than 9 and less than 14 and finally “high” for number of years 14 or higher. The results for the extended estimations are shown in Table 3.

**Table 3:** Marginal effects in Probit estimations on being in the labour force, 65–69 years old, separately for men and women, SHARE W5, Denmark, Germany and Sweden

	Men			Women		
	DK	DE	SE	DK	DE	SE
65 years	0.194*** (6.49)	0.241*** (8.80)	0.281*** (10.92)	0.123*** (5.41)	0.031*** (2.16)	0.208*** (11.19)
66 years	0.067*** (2.03)	0.176*** (6.21)	0.147*** (5.35)	0.063*** (2.71)	0.015 (1.00)	0.050*** (2.33)
67 years	0.060** (1.94)	0.113*** (3.83)	0.093*** (3.41)	0.033 (1.33)	−0.034** (−1.70)	0.055*** (2.69)
68 years	0.038 (1.10)	0.094*** (2.89)	0.123*** (4.53)	0.047** (1.96)	0.019 (1.05)	0.063*** (3.05)
Educ_low	−0.003 (−0.12)	0.042** (1.81)	−0.055*** (−3.05)	0.037*** (2.45)	0.012 (0.78)	−0.038*** (−2.61)
Educ_high	0.044** (1.89)	0.047*** (3.70)	−0.004 (−0.25)	0.093*** (5.96)	0.040*** (3.60)	0.030*** (2.46)
Self-assessed health	−0.037*** (−4.14)	−0.055*** (−8.87)	−0.027*** (−3.87)	−0.031*** (−5.01)	−0.026*** (−4.89)	−0.024*** (−4.79)
Pseudo R^2^	0.040	0.21	0.087	0.103	0.088	0.162
No. of obs	1765	2000	2273	1816	1935	2760

**Significance 5%, ***Significance 10%

For all countries and for both men and women, individuals aged 65 years have a significantly higher probability of labour force participation than the reference group of individuals aged 69 years. For Denmark this is also found for the 66 years old. For men in Germany and for both men and women in Sweden we find significant positive coefficients to all ages 65 to 68 years compared with 69 years.

The coefficients for educational levels confirm a prior expectation of higher labour force participation in the group with “high” education compared with the excluded group with “medium” education. The only exception to this pattern is found for men in Sweden. Here we find, however, a significant negative coefficient to “low” education. So, for Sweden, men with “high” and “medium” education have significantly higher participation probabilities, however without any distinction between “medium” and “high” education. For men in Germany and women in Denmark we find another pattern, i. e. both individuals with “low” and “high” education work with higher probability than those in the “medium” group. This could be interpreted as a reflection of “necessity” to work for the “low” educated and “preferences” for work in the “high” education group. An interpretation like this for low income groups in Germany is found in Eschelbach (2011) and Hochfellner (2013). Finally, we find a very clear gradient for women in Sweden, with the “low”educated working with significantly lower probability and the “high” educated working with a significantly higher probability than the medium group.

## Concluding remarks

In recent years a main emphasis in many OECD countries has been on rolling early retirement programs back. This has turned out to be supported by strong positive cohort trends in education and health among new generations of 60–64 years old. Our focus is on the “next” age group, the 65–69 years old. Labour force participation in this age group is highly interesting both in terms of the demographic challenge and in terms of major policy reforms announced or decided upon for this age group in the coming years.

We focus on this age group in Denmark, Germany and Sweden over the most recent decade. These countries have had different cyclical profiles in recent years, they have had different policy ambitions regarding labour market attachment for the 65–69 years old and the structure of their welfare states and labour markets differs. They share, however, to a large extent the same demographic challenge.

We find that labour force participation – which is identical to employment in this age group – has gone up in all three countries. The largest increase in labour force participation has taken place in Sweden following by Germany, while the increase in Denmark is rather small.

In line with previous research, our results suggest that changes in retirement policies, education and health contribute to explain the recent increase in employment rates among individuals aged 65–69 years. However, our results extend what we already know by showing that – although we focus on three rich OECD countries in Europe – the importance of changes in retirement policies and education in particular seem to differ depending on the country in focus.

As mentioned, main emphasis of policy reforms has been to roll back early retirement programs. In all three countries, however, there have been smaller policy changes directed towards the 65–69 years old group to create and strengthen incentives to postpone retirement, most explicitly in Sweden. Financial incentives seem most important in Germany and only of minor importance in Denmark, where policy changes directed towards individuals above the age of 65 appear to have been too small so far to affect retirement behaviour significantly.

Along with financial incentives in the social security system, education and health are two important determinants of labour force participation. In a shift share analysis, we find that about 25% of the increase in labour force participation among the 65–69 years old in Denmark and Sweden is explained by changes in education. This is found to be more important for women than for men in both countries. In Germany, changes in education appear to be less important.

For all three countries and for both men and women, we find increasing life expectancy and increasing number of expected healthy year at age 65. The latter in particular makes it more realistic to expect a significant labour force potential for the 65–69 years old group.

Our descriptions of the trends in important areas expected to influence labour force participation in the age group in focus is followed by a number of regression results relating labour force participation to age, education and health assessed by the respondents in wave 5 of the SHARE survey. Overall, we find the expected significant impact from a negative gradient in age, a positive impact from self-assessed health and a positive gradient in education. Entering single year age dummies and dummy variables for three levels of education we find significant differences by age. We find significantly higher labour force participation at ages 65, 66, 67 and 68 for men in Germany and for men as well as women in Sweden. The coefficients are declining as age 69 is approached but still indicate a significantly higher employment rate until 68 with 69 as the excluded group. This probably reflects the impact from the explicit policy measures directed at postponing retirement especially in Sweden. The educational dummy variables confirm the prior expected impact from “high” education. We find, however, also for two groups, men in Germany and women in Denmark, that both “low” educated and “high” educated work more than the intermediate group. A possible interpretation of this finding is that the “low” educated group to some extent postpone retirement out of financial necessity and the highly educated group continue working out of a preference for the type of work they are doing.

A final concluding point of prospective interest is that the 65–69 years old in the three countries experience labour force participation increasing most steeply in the post-2008 period of the great recession. This is the same surprising experience as found for the younger 60–64 years old group where recent cohorts also show steep increases in average education and health status.

## Figures and Tables

**Fig. 6:**
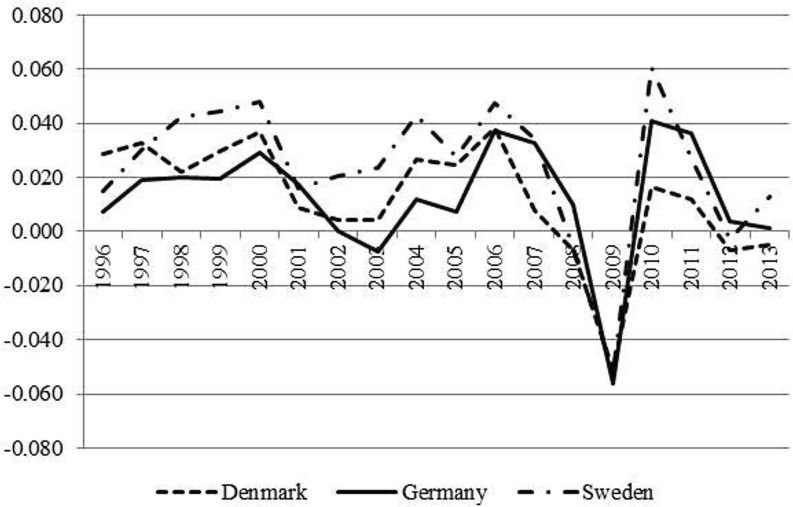
Real GDP annual growth. Denmark, Germany, and Sweden, 1996–2013

## Footnotes

1 We use the “sharew1_rel2-6-0” files for wave 1 and the “sharew5_rel1-0-0_imputations” file for wave 5.

2 For wave 1, we only have 187 observations for Denmark. The corresponding numbers for Germany and Sweden are 561 and 462 respectively. In the version of wave 5 we use, the number of observations is much larger. Part of the reason is that SHARE provides multiple imputations of the missing values. The number of multiple imputations is *m =* 5. The number of observations in wave 5 is 3935 for Germany, 5033 for Sweden and 3581 for Denmark.

3 However, results for Denmark based on register data suggest that labour force participation rates actually also do increase from around 2004 and on for these age groups in Denmark. However, the register data also suggest that the increase from 2004 to 2014 in Denmark is relatively small for both men and women compared to the increase for these groups in Germany and Sweden.

4 The lowest age for mandatory retirement was increased to 67 from 2001 for those who did not had a collective agreement stating another (lower) age and from 2003 for others.

5 Information about labour force participation rates by educational level is not available from EUROSTAT. However, it seems reasonable to assume that employment rates for individuals aged 65 to 69 years are identical to labour force participation rates as unemployment insurance is not available in the 65 and older group.

6 It is not obvious whether procedures for coding or real differences explain that the average number of years of schooling in Germany is about 1.5 years higher than in Denmark and Sweden.
